# Racial Ingroup Bias and Efficiency Consideration Influence Distributive Decisions: A Dynamic Analysis of Time Domain and Time Frequency

**DOI:** 10.3389/fnins.2021.630811

**Published:** 2021-05-10

**Authors:** Jiaxin Yu, Yan Wang, Jianling Yu, Jianmin Zeng

**Affiliations:** ^1^School of Applied Finance & Behavioral Science, Dongbei University of Finance and Economics, Dalian, China; ^2^Sino-Britain Centre for Cognition and Ageing Research, Faculty of Psychology, Southwest University, Chongqing, China

**Keywords:** racial ingroup favoritism, equity, efficiency, time-frequency analysis, ERP

## Abstract

Although previous studies have demonstrated that identity had effect on justice norms and behavioral decisions, the neural mechanism of that effect remains unclear. In this study, the subjects made their distributive decisions on the trade-off between equity and efficiency among Chinese and foreign children and their scalp potentials were recorded. Behavioral results showed that efficiency consideration played an important part in the distribution task. Meanwhile, participants gave preferential treatment to same-race children. Relative to the distribution within ingroup children, the distribution involving outgroup children induced higher N170 amplitude. The distribution involving outgroup children also elicited weakened P300 amplitude and enhanced delta response than the distribution within ingroup children when subjects are facing the conflict between equality and efficiency. In other words, ingroup bias affected the neural process of the trade-off between equality and efficiency. The combination of time-domain and time-frequency analyses provided spatiotemporal and spectral results for a better understanding of racial ingroup favoritism on distributive justice.

## Introduction

Imagine a violent and destructive earthquake hit an area, turning two villages into ruins. You, as a member of the rescue team, need to enter this area and provide food supplies to the two villages. However, because of the collapsed and blocked highways and roads, delivering food to both of the two villages would cause some meals to spoil. Therefore, you face such a choice dilemma: if you deliver the food to only one village to maximize the total amount of meals, it will be unfair for the other village; but if you want to help both villages, some food supplies will be spoiled, which are very precious at the moment. Meanwhile, you get the information that one of the villages was full of locals (the same nationality as you), and the other was full of immigrants. Are you more willing to help locals even if the other village gets nothing, or do you treat them equally even if several locals have to sacrifice? This dilemma illustrates the core issues of distributive justice, which involves trade-off among equity, efficiency, and identity. The current research aims to examine people’s decisions in such conflicts.

Humans show remarkable preferences for fairness and social equality. Evidence for the behavioral importance of the equity principle comes from a large body of behavioral economic experiments, such as the ultimatum game, the dictator game, and the public goods game (Camerer and Thaler, 1995; Hoffman et al., 1996; Levitt and List, 2007). Whereas most people generally prefer to promote equity in resource allocation, they may have to reconsider, sometimes, when equity comes at the expense of efficiency. Here we use the term efficiency as the sum of payoffs (Engelmann and Strobel, 2004). Engelmann and Strobel (2004) suggested that efficiency concerns was important in simple distribution experiments and the results turned out that efficiency had a major impact. Meanwhile, it had been shown that people were even willing to discard a resource over unequally allocating it (Shaw and Olson, 2012), because of the aversion of appearing partiality (Choshen-Hillel et al., 2015) or inequity responsibility aversion (Gordon-Hecker et al., 2017).

However, equity preference does not necessarily lead to fair behaviors. Individual choices depend not only on the type of preference but also on the economic, social, and cultural contexts and on the characteristics of the group with which they are interacting. The Social Identity Theory (SIT; Tajfel and Turner, 1979), which has been verified in typical social psychology studies, shows that people can form groups with only a slight excuse and start discriminating against other people not belonging to their group. One of the relevant implications, ingroup favoritism, also known as ingroup bias, is an effect indicating that people give preferential treatment to others who are perceived to be in the same group. Substantial evidences show that group identification can influence the concern of group members in behavior, attitude, and cognition (Brewer, 1979; Castano et al., 2002; Cikara and Van Bavel, 2014; Han, 2018). Particularly, some studies have focused on the effect of group bias on fairness norm enforcement. Researchers have found that hypnotic ingroup and outgroup suggestions can contribute to ingroup favoritism and outgroup rejection. Compared with the outgroup suggestion, the ingroup suggestion is associated with a significantly greater acceptance rate of unfair offers (Brüne et al., 2012). The group membership also modulates to offer evaluation at the processing stage, which shows that feedback-related negativity is more negative for extremely and moderately unequal offers versus equal offers in the ingroup interaction rather than in the outgroup interaction (Wang et al., 2017). Therefore, racial ingroup favoritism, which represents a socially constructed concept that categorizes humans into distinct groups based on shared social and cultural customs, may also have a consequence on distributive justice.

Furthermore, time-domain analysis and time-frequency analysis have gradually become impactful tools for assessing electrical and magnetic brain activity from event-related paradigms. To explore the neural process of underlying ingroup bias in distribution tasks, we focused on two event-related potential (ERP) components, N170 and P300. N170 is a negative polarity neuroelectric signal peaking at approximately 170 ms at posterior temporal recording sites and is larger over the right than left hemisphere. It is more negative to pictures of faces than other categories (Bentin et al., 1996). Furthermore, the N170 component can be influenced by social information, such as race and gender (Ito and Urland, 2005). Researches examining the racial ingroup bias in empathy reveal that the N170 component is decreased for other-race compared with same-race faces (Sheng et al., 2017; Han, 2018). By contrast, racial outgroup members are often viewed as threatening and therefore may elicit vigilant attention. Indeed, the N170 component is significantly enhanced in response to viewing black versus white faces for participants with strong pro-White bias (Ofan et al., 2011, 2013). These inconsistent findings suggest that the visual processing of race is malleable and depends on social motivations and contexts (Ofan et al., 2013; Amodio, 2014). Another ERP component, P300, is a positive deflection recorded by parietal electrodes at approximately 300 ms after stimulus onset. The P300 component has been reported in numerous social cognitive experiments, including social outcome evaluation and making decisions under complex social contexts (Chen et al., 2009; Wu et al., 2012). The P300 component reflects the conflict resolution process, and higher amplitudes are related to a higher proportion of allocated cognitive resources (van Dinteren et al., 2014; Zhu et al., 2018). In particular, previous studies show that the augmented P300 is found for self- and prosocial-relevant stimuli, which has a more important social significance, relative to control stimuli (Gray et al., 2004; Xiao et al., 2015). The time-frequency analysis is one of the most commonly used approaches to investigate the event-related oscillation, and its output includes changes in the oscillatory activity described in various frequency bands (Ergen et al., 2014). Recent investigations have demonstrated that the delta response contributes to P300 amplitude (Karakas et al., 2000; Ergen et al., 2008). The evoked delta response is suggested to be related to stimulus evaluation and decision making (Başar et al., 2001), and the larger delta response seems to be involved in a relatively easier decision process (Ergen et al., 2014). In addition, theta band activity is co-localized to the same area as hemodynamic signals stimulated by faces (Dravida et al., 2019). More importantly, theta band activity has been found to reflect the information contained in faces; for example, higher theta activity is reported for known faces compared with unknown faces (Güntekin and Başar, 2014). Those evidences suggest that theta band oscillations may be involved in face processing.

On the basis of previous research, the current study aimed to address the impact of racial ingroup and efficiency consideration on distributive decisions. We employed a distribution task which pitted equity against efficiency and ingroup. In each trial of a set of three children, participants had to decide who got the donation: the group of two children with equal donation or a single child. The option for a single child may either be more efficient (more total number of meals than the group of two children) or have no efficiency difference (equal total meals to the group of two children). Besides, the group of two children may be either typical Chinese-looking faces or typical Western-looking faces while the side of one child was a typical Chinese-looking face. The scenario of two Western children and one Chinese child was a trade-off between equity and ingroup. Participants had to choose between an option that was more equitable but for outgroup and an option that was for ingroup but less equitable. Informed by the available evidences reviewed above, we tested the following hypotheses. First, regarding the behavioral effects, we expected that participants were more likely to choose the group of two children due to inequity aversion; however, it could be declined in two conditions: one was the group of two children both foreigners due to ingroup bias, the other was the side of one child who was more efficient. Second, regarding the ERP effects, we expected to observe modulations of ERP components linked to social category and cognitive resource allocation, such as N170 and P300, in the process of dealing with different events containing social conflicts. We predicted to observe the difference in N170 between the two group types. For the mixed-group type, we predicted an even more negative N170 as the racial outgroup members could be viewed as threatening or competitors for limited resources in our design. Besides, we examined the P300 component, whose amplitude might be related to the amount of cognitive resources being used. We expected to find the P300 amplitude to be higher when equity and efficiency came into conflict since it might need more cognitive efforts. Moreover, the ingroup trials might be more self-relevant compared to mixed-group trials. Combining the above two points, we predicted the P300 amplitude to be higher when facing the trade-off between equity and efficiency within ingroup children than other scenarios. Third, the study utilized a time-frequency analysis to examine the differences in delta and theta oscillatory activities. We expected that delta and theta results would be in accordance with P300 and N170 results. If these hypotheses could be confirmed, then the results would reveal the cognitive processing of ingroup bias and trade-off.

## Materials and Methods

### Participants

Twenty-four healthy Chinese college students (nine females, age 21 ± 2 years, range 18–25 years) participated in this study for monetary compensation. Besides, all were right-handed with normal vision or corrected normal vision. None had any current or past psychiatric or neurological disease. The experiment lasted within 1 h, and each person received a payment of 50 yuan (about $7.7) for his/her participation. Written informed consent was obtained from each participant prior to the experiment. All methods were carried out in accordance with relevant guidelines and regulations, and this study was approved by the Administration Committee of Psychological Research of Southwest University.

### Stimuli and Procedure

At the beginning of the experiment, participants were required to read the brief introduction of the international Children Welfare, followed by an instruction on how to make their decisions. They were informed that a charity planned to provide extra nutritious meals to children in this international Children Welfare, and the quantity of meals for each child would be donated according to their decisions. Participants were highly emphasized that their choices would have a real impact on the gains for each child in the Children Welfare.

**FIGURE 1:**
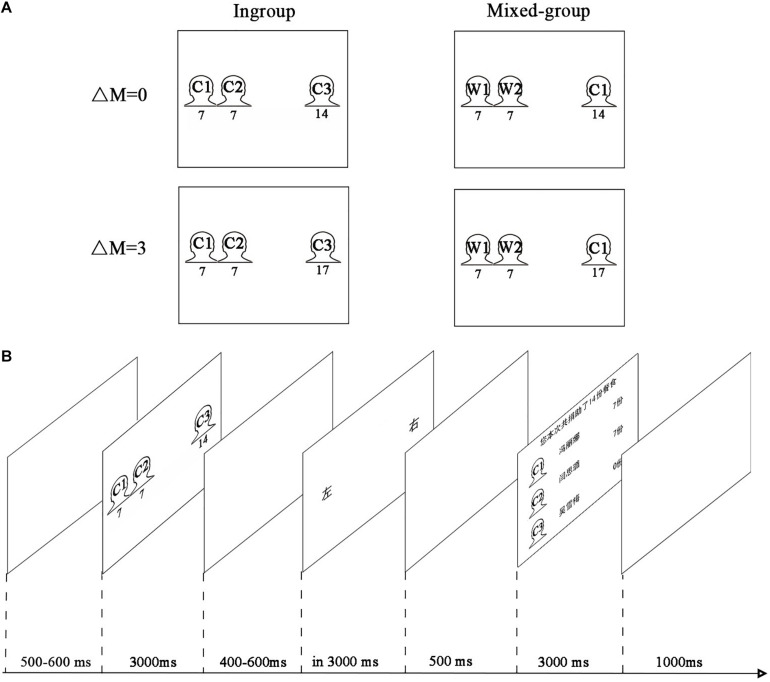
Experimental paradigm. **(A)** We presented four (2 × 2) kinds of stimuli. The first factor referred to ingroup type (ingroup: two Chinese children and one Chinese child; mixed-group: two Western children and one Chinese child). The second factor referred to efficiency difference between two options (ΔM = 0 meals vs. ΔM = 3 meal). Indicative instead of original images of children used here for a demonstration purpose (C represented Chinese child; W represented Western child). **(B)** Timeline of screens within a trial.

In each trial, participants decided which side could acquire the meals: the group of two children or a single child, with the position of the two options counterbalanced on the left and right sides of the screen. Participants had to make a trade-off between equity and efficiency or between equity and ingroup. The experiment had a 2 × 2 within-participant factorial design (Figure 1A). The first factor referred to group type (ingroup: two Chinese children and one Chinese child; mixed-group: two Western children and one Chinese child). The scenario of two Western children and one Chinese child was a trade-off between equity and ingroup. Participants had to choose between an option that was more equitable but for outgroup and an option that was for ingroup but less equitable. The second factor referred to efficiency difference between two options (ΔM = 0 vs. ΔM = 3). The option for a single child might be either more efficient (more total number of meals for a single child than the group of two children) or no efficiency difference (total meals for a single child were equal to those of the group of two children). In other words, participants must face the trade-off between equity and efficiency.

A total number of 120 trials were presented, and each condition contained 30 trials. Due to the single occurrence of each child’s face, 360 faces (240 Chinese children’s faces, 120 Western children’s faces) with marked racial differences were used in total. The gender of all images had been balanced, and each condition contained 15 trials of girls’ faces and 15 trials of boys’ faces. All faces were adjusted for luminance and contrast. After the rigorous selection, the presentations of three same-sex faces in each trial were matched for similar age, lighting condition, haircut, expression, and attractiveness. After that, five volunteers (two female) were asked to evaluate the emotional intensity of each photograph for four dimensions (i.e., happiness, sadness, anger, fear) on a 5-point Likert scale (1 = not at all, 5 = extremely strong). The perceived attractiveness was also evaluated on a 5-point Likert scale (1 = not at all, 5 = extremely attractive). Three hundred and sixty photographs were presented in random order. For each trial of three photographs, the repeated-measure analyses of variance (ANOVAs) were conducted on rating scores of emotional intensity and rating scores of perceived attractiveness, respectively. The ANOVAs did not show any significant effects (Ps > 0.1).

Participants were seated comfortably about 100 cm away from a personal computer screen in an electromagnetically shielded room. Presentations of trials were generated and controlled by the E-Prime software (Psychology Software Tools, Inc., version 1.1; Sharpsburg, Pennsylvania, United States). Four practice trials were administered before the formal test to help the participants be familiar with this task. The formal experiment process was shown in Figure 1B. In each trial, participants were firstly required to gaze at the center of the blank screen for 500–600 ms (uniformly distributed). Then, the distribution task was shown on the screen for 3,000 ms, which showed the children’s photographs, two children on one side and one child on the other side. The amount of meals appeared below the photograph of each child. The decision screen was presented after a blank screen of random duration (uniformly distributed within 400–600 ms), then participants were required to make their decisions by pressing a bimanual button (“left” or “right”) in 3 s. After the decision-making, participants would see a blank screen for 500 ms and then would observe a feedback screen with the amount of donated meals for each child for 3 s. Another trial would follow after a blank screen which lasted for 1 s. Participants were encouraged to minimize eye movements during EEG recording.

### Event-Related Potential Recording and Data Analysis

The electroencephalogram (EEG) was continuously recorded from 64 scalp sites using Ag/AgCl electrodes mounted on an elastic cap (Brain Product GmbH, Munich, Germany), according to the criteria of the international 10–20 system. All electrode recordings were referenced to the center between Fz and Cz and digitized at 500 Hz. Impedances were kept below 5 KΩ for scalp recordings and below 10 KΩ for EOG recordings. Band-pass and notch filtering (0.05–80, 50 Hz) were applied.

Preprocessing of EEG data was performed using EEGLAB (Delorme and Makeig, 2004) in the MATLAB environment (MathWorks). The data was referenced with the averaged amplitudes of the left and right mastoids. Continuous EEG data was band-pass filtered between 1 and 30 Hz. In addition, independent component analysis (ICA; Bell and Sejnowski, 1995) was performed on data of a single participant to identify and remove eye movements and eye blinks. The individual components which were screened for maps with a symmetric frontal topography, accounting for eye blinks and eye movements were discarded from further analysis. The analyzing epoch was time-locked to the onset of the distribution screen including children’s faces and the amount of meals. EEG epochs were extracted with a time window of 1,200 ms (200 ms pre-stimulus and 1,000 ms post-stimulus) for independent component decomposition. Trials containing other artifacts (peak-to-peak deflection exceeding ± 100 μV) were excluded from averaging. On average, 1.63% (SD = 4.01) of epochs were rejected.

We investigated relevant brain regions associated with differences in N170 and P300 on the basis of the previous studies and the observations from the grand-averaged ERP waveforms (Figure 3). Due to the fact that N170 is most appropriately measured at lateral parieto-occipital sites (Rossion and Jacques, 2008; Almeida et al., 2016), in the current study, the N170 mean amplitude was analyzed with four electrode sites (P5 and P7 for the left hemisphere, P6 and P8 for the right hemisphere) in the time window of 140–200 ms. Previous studies have found that P300 appears in the parietal lobe and usually peaks at the midline electrodes on the scalp (Delplanque et al., 2004; Wu and Zhou, 2009; Long et al., 2018). To explore the P300 effect in the present paradigm, the mean amplitude of P300 was measured in the 200–600-ms time window at CPz, Pz, and POz.

Additionally, the Morlet wavelet for time-frequency analysis of ERP data was also applied in this study. The continuous wavelet transform is a multiresolution analysis technique that provides a good compromise between time and frequency resolution. To represent the oscillatory activity that is phase-locked to stimulus onset (the evoked activity), the wavelet transform is computed on average of the single trials. A wavelet is a simple oscillating amplitude function that is localized in time, and in this general form it is referred to the mother wavelet (Samar, 1999). To create TF signal representations, the mother wavelet is systematically stretched and reduced in time to be sensitive to lower- or higher-frequency activity, respectively. It is necessary to set the bandwidth and center frequency discreetly to define a mother wavelet (Zhang et al., 2017). In the current study, the bandwidth and center frequency were both suitably set to 1 in defining the mother wavelet. The potential activities of regions of interest (ROIs) were measured in a 1–30-Hz frequency window. The conventional rectangle method was used to mark the region of evoked event-related oscillation (Zhang et al., 2017, 2020). Figure 4 depicted the time-frequency results of phase-locked ERP, where delta oscillation (1–4 Hz) in the time window of 200–400 ms and theta oscillation (4–7 Hz) in the time window of 140–260 ms were clearly observed. In order to test the effects of group type and efficiency difference on theta and delta responses, we calculated the power of delta band with three electrode sites (CPz, Pz, and POz) and the power of theta band with four electrode sites (P5, P6, P7, and P8).

### Statistical Design

All analyses were performed using IBM SPSS Statistics for Windows, version 16.0 (IBM Corp., Armonk, NY, United States). Behavioral results in terms of mean percentage of choosing two children and mean response time were computed separately by each participant. The behavioral results as well as the time-domain components of N170 and P300 and the time-frequency components for delta and theta responses were conducted separately by repeated-measure analyses of variance (ANOVAs), having group type (ingroup vs. mixed-group) and efficiency difference (ΔM = 0 vs. 3) as two within-subject factors. *P*-values were corrected using the Greenhouse–Geisser method. The level of significance was set at *p* = 0.05. When appropriate, *post hoc* tests were performed. Partial eta-squared was reported to demonstrate the effect size of the statistical results.

## Results

### Behavioral Results

**FIGURE 2:**
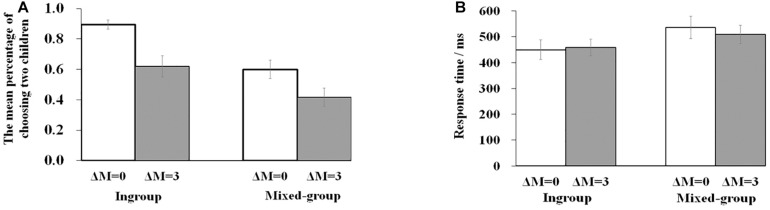
**(A)** The mean percentage of choosing two children for four (2 × 2) kinds of stimuli (ingroup and ΔM = 0; ingroup and ΔM = 3; mixed-group and ΔM = 0; mixed-group and ΔM = 3). **(B)** The mean response time for four (2 × 2) kinds of stimuli. Bars represented standard error. Levels of significance:*p < 0.05; **p < 0.01; ***p < 0.001.

Table 1 presents the converging results of the percentage of choosing two children and the response time. The percentage of choosing two children demonstrated a significant effect of group type [*F*(1,23) = 28.712, *p* < 0.001, η_p_^2^ = 0.555]. *Post hoc* tests revealed that the rate of choosing two ingroup children was significantly larger than choosing two outgroup children (0.757 vs. 0.508, *p* < 0.001). The main effect of efficiency was also significant [*F*(1,23) = 23.355, *p* < 0.001, η_p_^2^ = 0.504), with a smaller percentage of choosing two children in the condition of ΔM of three than in the condition of ΔM of 0 (0.518 vs. 0.747, *p* < 0.001). The interaction between group type and efficiency failed to reach a significant level [*F*(1,23) = 1.445, *p* = 0.242, η_p_^2^ = 0.059]. Figure 2A summarizes the percentage of choosing two children for four kinds of scenarios.

**TABLE 1:** Summary of behavior results on percentage of relatively equity decisions and response time (M ± SE).

	Percentage of choosing two children			Response time (ms)		
	ΔM = 0	ΔM = 3	ALL	ΔM = 0	ΔM = 3	ALL
Ingroup	0.89 ± 0.03	0.62 ± 0.07	0.76 ± 0.04	449.90 ± 38.48	458.52 ± 32.60	454.21 ± 34.96
Mixed-group	0.60 ± 0.06	0.42 ± 0.06	0.51 ± 0.05	536.24 ± 43.20	509.23 ± 35.39	522.74 ± 38.52
ALL	0.75 ± 0.04	0.52 ± 0.06	0.63 ± 0.04	493.07 ± 39.71	483.87 ± 32.45	488.47 ± 35.66

Repeated-measure ANOVA on response time revealed a significant result on group type [*F*(1,23) = 14.417, *p* = 0.001, η_p_^2^ = 0.385]. The response time of the ingroup was shorter than the mixed group (454.210 vs. 522.736 ms, *p* = 0.001). The interaction between group type and efficiency reached a marginal significant level [*F*(1,23) = 4.173, *p* = 0.053, η_p_^2^ = 0.154]. *Post hoc* tests revealed that the response time of the ingroup was shorter than the mixed group not only in the condition with efficiency difference (*p* < 0.001) but also under the condition with no efficiency difference (*p* = 0.021). There was no significant main effect of efficiency [*F*(1,23) = 0.487, *p* = 0.492, η_p_^2^ = 0.021]. Figure 2B summarizes the response time for four kinds of scenarios.

### Time-Domain Results

#### N170

**FIGURE 3:**
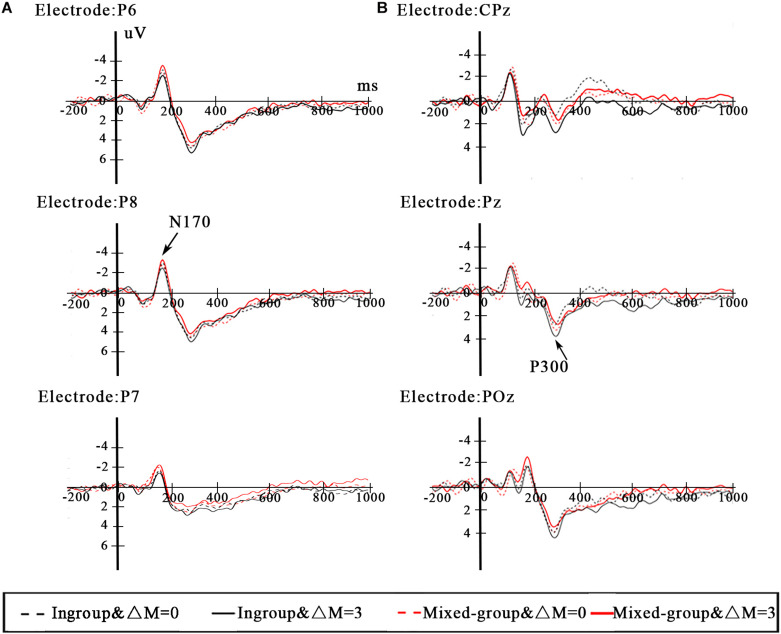
**(A)** ERP waveforms from posterior electrodes P6, P7, and P8 for N170. **(B)** ERP waveforms from parietal electrodes CPz, Pz, and POz for P300.

The repeated-measure ANOVA on the N170 mean amplitude revealed a significant main effect of group type [*F*(1,23) = 5.044, *p* = 0.035, η_p_^2^ = 0.180]. *Post hoc* tests revealed that the N170 amplitude elicited by the ingroup was lower than the mixed group (*p* = 0.035). There was neither significant main effect of efficiency [*F*(1,23) = 0.010, *p* = 0.921, η_p_^2^ < 0.000] nor interaction between group type and efficiency [*F*(1,23) = 2.332, *p* = 0.140, η_p_^2^ = 0.092]. The grand average ERPs at the P6 and P8 electrodes are depicted in Figure 3A.

#### P300

The results of repeated-measure ANOVA for the P300 mean amplitude yielded a significant interaction between group type and efficiency [*F*(1,23) = 5.285, *p* = 0.031, η_p_^2^ = 0.187]. *Post hoc* tests revealed that the P300 amplitude of ΔM = 3 was also larger than ΔM = 0 (*p* = 0.004) when dealing with distributions between the same ethnic group members. However, when dealing with distributions between mixed-group members, the P300 amplitude of ΔM = 3 and ΔM = 0 had no difference (*p* = 0.647). The P300 amplitude elicited by the ingroup was larger than the mixed group (*p* = 0.042) under the condition of ΔM = 3. However, under the condition of ΔM = 0, the P300 amplitude showed no difference between ingroup and mixed group (*p* = 0.384). The main effect of efficiency reached a marginal significant level [*F*(1,23) = 3.255, *p* = 0.084, η_p_^2^ = 0.124], and the P300 amplitude elicited by ΔM = 3 was larger than ΔM = 0. There was no significant main effect of group type [*F*(1,23) = 0.249, *p* = 0.623, η_p_^2^ = 0.011]. The grand average ERPs at the CPz and Pz electrodes are depicted in Figure 3B.

### Time-Frequency Results

#### Delta Frequency Band

**FIGURE 4:**
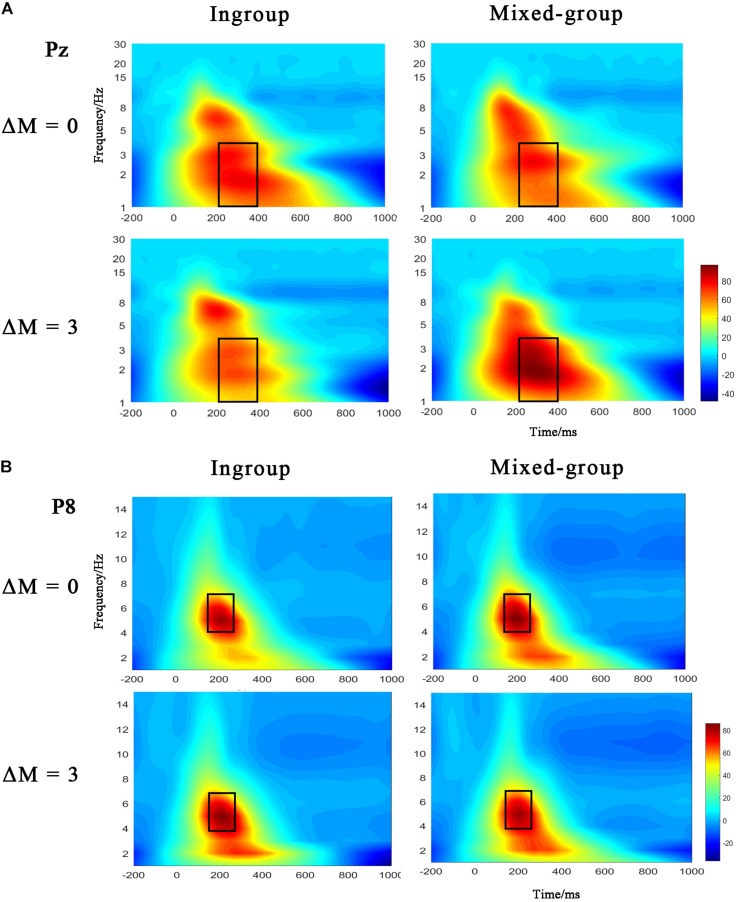
**(A)** Time-frequency representation of ERP signals at the electrodes Pz for delta activity (1–4 Hz). **(B)** Time-frequency representation of ERP signals at electrodes P8 for theta activity (4–7 Hz). Dark rectangles marked the time/frequency window used in the statistical analysis.

The ANOVA applied to delta response showed the statistical significance of the interaction between group type and efficiency [*F*(1,23) = 4.512, *p* = 0.045, η_p_^2^ = 0.164]. The *post hoc* test revealed that increased delta power was slightly enhanced by the mixed group than ingroup (*p* = 0.070) under the condition of ΔM = 3. There was neither significant main effect of group type [*F*(1,23) = 0.649, *p* = 0.429, η_p_^2^ = 0.027] nor main effect of efficiency [*F*(1,23) = 0.003, *p* = 0.961, η_p_^2^ < 0.001]. The delta oscillation at Pz electrode is depicted in Figure 4A.

#### Theta Frequency Band

The evoked theta response was clearly located in the occipital-temporal region (Figure 4B). However, the repeated-measures ANOVA for theta oscillations showed no significance, either on the main effect of group type [*F*(1,23) = 0.546, *p* = 0.467, η_p_^2^ = 0.023] or on the main effect of efficiency [*F*(1,23) = 0.970, *p* = 0.335, η_p_^2^ = 0.040), or on the interaction between group type and efficiency [*F*(1,23) = 0.752, *p* = 0.395, η_p_^2^ = 0.032].

## Discussion

The purpose of the present study was to explore the neural basis of racial ingroup bias on distributive justice which concerns how individuals and societies distribute benefits in a just or moral way. Hence, we designed a distribution task that took racial identities into account. As expected, the percentage of choosing the two-children side declined in the distribution involving outgroup children. In line with previous studies, it supported the social identity theory, suggesting that people are giving preferential treatment to others when realizing they are in the same racial group. Besides, extra meals for the side of one child decreased the percentage of making relatively equitable choices. Furthermore, we used the time-domain and time-frequency analyses to explore the neural mechanism of ingroup effects on distributive justice. The existence of other-race faces elicited the enhanced N170 component. Making decisions between ingroup children elicited higher P300 amplitude than decisions involving outgroup children when facing the conflict between equity and efficiency, which was also compatible with the results of the delta response. Besides, the existence of efficiency variation elicited higher P300 amplitude in the distribution not involving outgroup children.

During this allocation experiment, donating to two children was regarded as a relatively equitable decision, whereas the efficiency accounted for a larger part in the two competitive sides. Participants must weigh both equity and efficiency of the distribution. In addition, the racial in/outgroup factor was also considered because the group of two children might be from China or West, which could be easily recognized from their photographs. In the scenario involving outgroup children, participants must make a trade-off between equity and ingroup.

With respect to the behavioral data, distributive justice was influenced by equity, efficiency, and ethnic factor. The percentage of choosing two children was significantly lower when one child obtained more extra meals (ΔM = 3) compared with the situation when one child obtained the same amount of meals as those of the other two children (ΔM = 0). Moreover, the percentage of making relatively equitable decisions in the distribution involving ingroup children, on average, was up to 75.7%. However, the proportion of donating to two foreign children in mixed-group trials dropped significantly to 50.8%. Therefore, ingroup favoritism or ingroup bias should be highly valued in the trade-off between equity and efficiency. The response time variation within the group types provided another perspective for ingroup bias. The results of response time showed that the distribution between ingroup children was significantly faster than the distribution involving outgroup children. This result may imply that the course of recognizing faces and/or dealing with distributions involving mixed race was a time-consuming neurological process.

The N170 component was evoked in response to the face array in our study. Previous research had shown that N170 amplitude was elicited by the face array at encoding which increased as the number of faces increased by using the array consisting of one to four faces (Morgan et al., 2008). As task affected N170’s sensitivity to race, Senholzi and Ito (2013) had demonstrated that White perceivers focusing at the level of individual identity showed larger N170s to racial outgroup Black than ingroup White faces, but when focusing at the level of racial category, N170s were larger to ingroup White than outgroup Black faces, and no effect of race on N170 was found when focusing on the distinction between faces and non-faces. In our study, greater N170 amplitude was elicited by mixed-group versus ingroup. One possible explanation was that subjects might focus at the level of individual identity in the distribution task. On the other hand, previous studies had confirmed that the visual processing of race was malleable and depended on social motivations and contexts (Caldara et al., 2004; Walker et al., 2008; Brebner et al., 2011; Amodio, 2014), which resulted in inconsistent results of N170 by race. As seen in the studies by Sheng and Han (2012) and Sheng et al. (2017), the face-sensitive N170 was weaker in response to racial outgroup faces than racial ingroup faces. In these tasks examining the racial ingroup bias in empathy, subjects empathized the pain of outgroup members to a less degree relative to racial ingroup members. In contrast, the N170 component was significantly enhanced in response to viewing black versus white faces for participants with strong pro-White bias (Ofan et al., 2011, 2013). In these contexts, outgroup members might be viewed as threatening and therefore might elicit vigilant attention. The fMRI studies also suggested that amygdala activation reflected an immediate or implied threat response to racial outgroup members (Kubota et al., 2012; Chekroud et al., 2014; Molenberghs and Louis, 2018). In our study, outgroup members were perceived as threats due to their roles as potential competitors for limited resources, therefore greater engagement was required for the facial encoding processes in the scenario of involving outgroup children than the scenario of three ingroup children. These were consistent with the current result that enhanced N170 was found for the mixed group than the ingroup.

From the perspective of electrophysiology, racial ingroup membership had a substantial influence on the trade-off between equity and efficiency, as reflected by the well-known P300 component. It was found that making decisions between ingroup children elicited a more positive P300 component than making decisions involving outgroups when facing the conflict between equity and efficiency. The P300 component was reported in advanced cognitive processes under social contexts, such as evaluation and stimulus categorization, memory encoding and updating, and decision-making (Chen et al., 2009; Fishman et al., 2011; Zhang et al., 2012; Gyurovski et al., 2018; Pfabigan et al., 2019). The closest to our study would be that of Chen et al. (2009) which tested Chinese participants who had experienced the Sichuan earthquake in May 2008. Those participants were presented with name-pairs that referred either to relatives (e.g., father–mother) or to strangers (e.g., stranger A–stranger B) and were asked to decide which of the two people they would rescue in case of an earthquake. In this social dilemma task, a larger P300 was observed when the choice was between two relatives which contained much stronger dilemma conflicts rather than between two strangers, which suggested that P300 might be related to the dilemma interference resolution processes—modulating and controlling cognitive conflict. In our study, the distribution with ingroup children meant that participants must face the dilemma interference between equality and efficiency. However, for distribution involving outgroup children, participants would be free from the dilemma of equity and efficiency due to racial ingroup favoritism, and individuals might be more likely to choose one ingroup child with more extra meals compared with two outgroup children with less meals. Compared with the distribution involving the outgroup, the trade-off between equity and efficiency might require more cognitive efforts in distribution between ingroups, which elicited enhanced P300 amplitude. In addition, when making decisions in the scenario of all ingroup children, the P300 amplitude was larger when participants had to weigh equity and efficiency (ΔM = 3) than when they only need to consider equity with no efficiency variance (ΔM = 0). The social economic decision-making could be understood as a dual-system process, integrating the influence of deliberative and affective subsystems, which might activate several areas including anterior cingulate cortex and the insula (Hsu et al., 2008; Harlé and Sanfey, 2012; Zinchenko and Arsalidou, 2018). In particular, the anterior cingulate cortex was shown to activate to the detection of distributional inequity in economic tasks, which could be explained as a violation of social norms, and the insula might be associated with a generic sense of cognitive demand related to the inequity encoding (Hsu et al., 2008; Zhong et al., 2016; Zinchenko and Arsalidou, 2018). Accordingly, the current results were in favor of the above hypothesis, and subjects would be conscious of much stronger dilemma conflicts when making a trade-off between equity and efficiency, which might result in greater P300 compared to making decisions with no efficiency variance. A similar result was found in the scenario of ingroup children but did not survive in the scenario of involving outgroup children. Ingroup favoritism increased the percentage of choosing the ingroup side, which might reduce the conflict between equity and efficiency itself.

The time-frequency analysis revealed the activations of the delta and theta bands during the experiment. The occipital–temporal distributed theta power (4 to 7 Hz) was elicited in the 140–260 ms time window in our experiment, which was generally in agreement with those on face recognition task in the literatures (Zion-Golumbic et al., 2010; Güntekin and Başar, 2014). With regard to a recent study, theta band oscillations were localized to the right occipital face area, where the expected N170 response was observed for faces (Dravida et al., 2019). Although N170 mean amplitude revealed a significant main effect of group type, event-related oscillations in the theta frequency range seemed to be hardly related to our experimental design. The theta frequency as the early synchronization was tentatively interpreted as an indicator for activation of the visual system, which was connected with recognition of physical parameters in a visual image (Başar, 1999; Yang et al., 2011). Therefore, the information contained in the theta band might be related to face perception rather than social category processing which needed to be explained by further research. At 1–4 Hz, the evoked delta response was significantly influenced by the interaction of group type and efficiency difference. The evoked delta response is suggested to be related to stimulus evaluation and decision making (Başar et al., 2001), and the larger delta response in the congruent condition seems to involve a relatively easier decision process (Ergen et al., 2014). The time-frequency results of the current study were supported by these recent findings about stimulus processing. When the side of a single child got more extra meals, the distribution not involving the outgroup compelled participants to weigh equity and efficiency. For distribution involving outgroup children, participants would be free from the dilemma of equity and efficiency due to racial ingroup favoritism which was a relatively easier condition that generated a larger evoked delta band.

More broadly, our results potentially shed light on the racial effects on central problem of distributive justice: the trade-off between equity and efficiency. The functional magnetic resonance imaging (fMRI) study on distributive justice provides neural spatial evidence that the brain encodes the inequity region and efficiency region separately, the putamen responds to efficiency, whereas the insula encodes inequity, and the caudate/septal subgenual region encodes a unified measure of efficiency and inequity (utility) (Hsu et al., 2008). Using the ERP technique, our finding investigates the time course of the distributive justice that the conflicts between equity and efficiency require a larger amount of cognitive resources engaged in processing a given scenario. Another contribution of our study is to show that ingroup bias besides efficiency consideration plays an important role in distributive decisions. The early face perception component responses to race and greater N170 amplitudes are elicited by mixed group versus ingroup. The ingroup favoritism may affect the neural process of the trade-off between equality and efficiency, and it modulates the P300 amplitude and delta power. As with our work, a key goal of all those relevant researches is to improve our understanding of the determinants of fair behavior.

However, the current study is the preliminary one to investigate the neural mechanism of distributive justice involving equity, efficiency, and in/outgroup factors, which has a few limitations. Firstly, this study is generally based on the perspective of Chinese participants and stimuli. Future studies should also include Western samples to examine whether the effects reported here are universal. Similar investigations for other age groups from various industries are also expected to be seen. Secondly, the present study enhances our understanding of distributive justice involving ingroup bias and the neural responses to such social conflicts. Reducing racial bias in allocation decisions through the manipulation of cognitive strategies awaits future studies in conjunction with fMRI or transcranial direct current stimulation (tDCS).

## Conclusion

This study investigated the underlying neural mechanisms of racial ingroup bias in the trade-off between equity and efficiency through time-domain analysis and time-frequency analysis. These two approaches interpreted the existence of racial ingroup bias in different ways, both testifying the influence of racial identity and efficiency consideration on equitable choices. Our study provided evidence that the ingroup preference affected the neural process of the trade-off between equality and efficiency and resulted in weakened P300 amplitude and enhanced delta activation. The present findings may provide new insights into the neural mechanism of real-life social behaviors on distributive justice and ethnic prejudice.

## Data Availability Statement

The datasets generated for this study are available on request to the corresponding author.

## Ethics Statement

The studies involving human participants were reviewed and approved by the Administration Committee of Psychological Research of Southwest University. The participants provided their written informed consent to participate in this study.

## Author Contributions

JY performed the data analysis and drafted the manuscript. YW developed the study concept and contributed to the study design and data collection. All the authors provided the critical revisions and approved the final version of the manuscript for submission.

## Conflict of Interest

The authors declare that the research was conducted in the absence of any commercial or financial relationships that could be construed as a potential conflict of interest.
